# The consequence of head-loading on the neuro-musculoskeletal health of the ILembe District youth of KwaZulu-Natal

**DOI:** 10.4102/ajod.v11i0.1039

**Published:** 2022-12-14

**Authors:** Tebogo G. Motaung, Terry J. Ellapen, Yvonne Paul

**Affiliations:** 1 Department of Sport Rehabilitation and Dental Science, Faculty of Science, Tshwane University of Technology, Pretoria, South Africa

**Keywords:** head-loading, proprioception, neuromusculoskeletal health, youth, biokinetics, pain

## Abstract

**Background:**

Head-loading, as a mode of transporting food, water and firewood, is a longstanding tradition assigned to female South African youth and has been associated with adverse health consequences.

**Objectives:**

This study determined the impact of head-loading on the neuromusculoskeletal health and proprioception of female South African youth.

**Method:**

This study comprised a counterbalanced, within-subject, single-factor experimental design which compared the changes that occurred when the same independent variable (head-loading) within two homogenous groups was measured in terms of the dependent variables (outcomes: neuromusculoskeletal pain and proprioception) at two time periods, before and after the introduction of the independent variable. A cohort of South African female youth (*n* = 100), aged 9–17 years, voluntarily partook in the study. The participants were randomly distributed into an experimental (*n* = 50) and a control (*n* = 50) group. The experimental group stood in a head-loaded state with their respective habitual head-load mass. Their proprioception measurements were compared during their unloaded versus loaded states, with the proprioceptive measurements including the total proprioception index, the anterior–posterior (front–back) index and the medial–lateral (side-to-side) index. Participants furthermore completed a head-loading health-related questionnaire.

**Results:**

Participants had a mean age of 12.3 ± 2.5 years, body mass of 44.4 ± 13.7 kg, stature of 145 ± 10 cm and a head-load mass of 8.0 ± 2.5 kg. Participants had poorer medial–lateral proprioception during head-loading as compared to their unloaded state (1.4 ± 0.8 as compared to 1.6 ± 0.9) (*p* < 0.05). Most youth (96%) experienced neuromusculoskeletal pain in their cervical vertebrae (40.9%), shoulders (27.3%), lumbar vertebrae (10.7%), arms (8.3%), legs (8.3%), knees (1.9%), fingers (1.5%), toes (0.5%) and thoracic vertebrae (0.5%) (χ^2^: *p* < 0.05).

**Conclusion:**

Head-loading adversely affects the medial–lateral proprioception and neuromusculoskeletal health of participants.

**Contribution:**

The findings of this study confirms that head-loading produces musculoskeletal pain.

## Introduction

Head-loading is a longstanding traditional mode of transporting food, water and firewood which is practised in many rural South African communities (Kurten et al. 2020; Porter et al. 2013). This important domestic occupational responsibility is allocated to female pubescents and adolescents and is associated with long-term neuromusculoskeletal injury and disability (Echarri & Forriol 2005; Porter et al. 2013). Young girls and women carry head-load masses ranging from 2 kg to 35 kg, transporting them over distances varying from 2 km to 10 km (Kurten et al. 2021b; Porter et al. 2013); the consequences of habitual head-loading are both positive and negative (Kurten, Ellapen & Paul 2021a). Porter et al. (2013) reported that female youth who habitually carry head-loads and assist with routine domestic chores are considered to be contributing members of the family; consequently, this causes an exponential increase in their wedding dowry (Kurten et al. 2020). The negative health impact of habitual head-loading includes both acute and chronic neuromusculoskeletal pain, intervertebral disc prolapse and spondylolisthesis (Echarri & Forriol 2002, 2005; Ellapen et al. 2009; Porter et al. 2013).

Habitual head-loading can be viewed as an occupational task that many rural female youth perform (Kurten et al. 2020; Porter et al. 2013). Furthermore, while many Africans and Asians work as porters, carrying external loads on their heads (Porter et al. 2013), Asian porters employ unique equipment for head-loading that includes a *namlo* [tumpline] connecting the forehead to a *doko* [basket], which rests against the porter’s back and may occasionally be rested on a *tokma* [T-shaped stick], which is also used as an alpenstock (Bastien et al. 2016; Minetti, Formenti & Ardigo 2006). Porter et al. (2013) reported that the most popular method of external load carriage in Africa is a derivative of the Asian head-loading technique. African porters place a soft piece of cloth upon their heads which is twisted tightly and then manipulated to form a circle in order to both stabilise and provide cushioning for the load (Lloyd et al. 2010).

While habitual head-loading porterage among rural South Africans is primarily considered a domestic occupational task (Kurten et al. 2021a), many African and Asian individuals carry external loads in cities and rural areas as professional porters (Minetti et al. 2006; Porter et al. 2013). Epidemiological investigations among African head-loading porters have reported an incidence of spondylolisthesis and intervertebral disc compression as well as symptoms of neuromusculoskeletal pain and persistent fatigue (Echarri & Forriol 2002, 2005; Ellapen et al. 2009). Echarri and Forriol (2002) reported a 20% incidence of spondylolisthesis, a 25.8% incidence of intervertebral disc prolapse, a 35.3% incidence of anterior osteophyte development and a 9.4% incidence of posterior osteophyte development among Congolese female porters. Ellapen et al. (2009) reported that South African adolescent female porters commonly experience musculoskeletal pain in their vertebrae (73.6%), shoulders (21%) and arms (5.2%). Geere, Hunter and Jagals (2010) further reported that South African adolescents who habitually head-load external masses experience a high incidence of cervical vertebral (69%) and lumbar vertebral (38%) neuromusculoskeletal pain.

While the impact of head-loading on the proprioception capabilities of South African female youth porters has not yet been the subject of investigation, the proprioception capabilities of Asian head-loaders have been investigated (Hoque, Grangeon & Reed 1999; Hoque et al. 2012). Hoque et al. (1999, 2012) reported an association between head-loading and poor proprioception, incurring a high incidence of falls and injuries. It is important to note that the Asian method of head-loading employed by the porters in these studies differs from the African method. Nevertheless, a parallel health-related investigation should be conducted so as to validate these findings among an indigenous South African cohort. Insofar as proprioception enables an individual to be better orientated within their environment, increasing their stability (Prentice 2015), poor proprioception has been related to an increased risk and incidence of injuries (Han et al. 2015; Prentice 2015).

The practise of head-loading is a rich part of the African culture and social infrastructure, which will be extremely difficult to terminate (Echarri & Forriol 2002, 2005; Ellapen et al. 2009; Geere et al. 2010), and Kurten et al. (2020) furthermore reported that community elders in rural South African villages are reluctant to allow female pubescents and adolescents to change their method of transporting external loads. Many rural community elders feel that only men should be allowed to use animals, animal-drawn vehicles and motorised vehicles to transport external loads. The transporting of water, firewood and food is considered to be the responsibility of pubescent and adolescent girls (9–17 years), who must use the head-loading method (Kurten et al. 2020; Potgieter, Pillay & Rama 2018). More research confirming the adverse health effects of head-loading can be used to persuade rural community elders to rethink their mindset and allow pubescent and adolescent girls to change their method of transporting food, firewood and water. From a scientific perspective, the evidence documented by Ellapen et al. (2009) and Geere et al. (2010) that daily head-loading produces cervical, thoracic and lumbar vertebral pain among pubescent and adolescent South Africans needs to be validated. To this end, Thomas, Nelson and Silverman (2015) recommended the validation of previous novel research in order to create interest in a given field of study. That said, the effect of head-loading on the proprioception of South African pubescents and adolescents has not yet been investigated. Thus, this study represents original research, which strengthens the rationale for the study. The findings of this study will increase public awareness and significantly augment the currently limited body of evidence, which in turn can help medical doctors, physiotherapists and biokineticists to develop strategies to rehabilitate – and/or prevent – neuromusculoskeletal pain and injuries among pubescents and adolescents who carry head-loads. This study determined the impact of head-loading on the neuromusculoskeletal health and proprioception of female South African youth.

## Methods

### Research design

In this study, a counterbalanced within-subject single-factor experimental design was used to compare the changes that occurred when the same independent variable (head-loading), within two homogenous groups, was measured against the dependent variables (outcomes: neuromusculoskeletal pain and proprioception) at two time periods, before and after the introduction of the independent variable (the experimental manipulation) (Passer 2017; Price, Jhangiani & I-Chant 2015; Thomas et al. 2015). The researcher used a homogenous group of participants who could head-load external masses. The intention of the study was to determine the impact of head-loading on a group of homogenous participants who regularly carry head-loads. This study also sought to validate the earlier findings of the study by Ellapen et al. (2009) and Geere et al. (2010) conducted in South Africa, insofar as these were novel papers in the field of rehabilitation. The unloaded group served as a control group, given that head-loading was the intervention (Passer 2017; Price et al. 2015), with the experimental group experiencing the head-loading (intervention).

**FIGURE 1:**
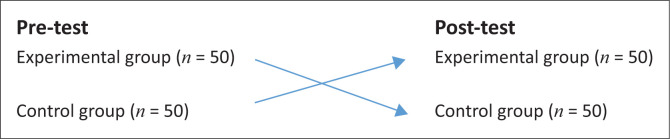
A counterbalanced within-subject single-factor experimental design.

Furthermore, the implementation of a counterbalanced within-subject single-factor experimental design allowed for randomisation of participants, which resulted in a pretest–post-test experimental research design. Randomisation into the head-loaded (experimental) group or the unloaded (control) group during the pretest stage was made by the participants’ selection of a ball from a hat: unknown to the participants, the selection of an orange ball placed the participant in the head-loading (experimental) group during the pre-test stage of the study, whereas the selection of a white ball placed the participant in the unloaded (control) group in the pretest phase (Figure 1).

The inclusion criteria of the study were that participants had to head-load daily, that they were between the ages of 9 and 17 years and of female gender and that their participation was voluntary. The reason for the delimitation of the cohort’s age to 9–17 years was based on literature reporting that the transportation of water, firewood and foods is considered to be the responsibility of pubescent and adolescent girls (9–17 years), who are required to use the head-loading method of transportation (Geere et al. 2010; Kurten et al. 2021b; Potgieter et al. 2018). The mass of participants’ habitual head-loads and their body mass were measured on an electronic scale. Demographic characteristics including age, body mass and stature were recorded.

### Sample size

The sample size was calculated using Cochran’s formula to ensure power of significance of the study findings, which was calculated to be 95.45 and can be approximated to 100 for ease of experimental design. The experimental (*n* = 50) and control (*n* = 50) groups were established through participants’ personal selection of a white (control) or orange ball (experimental) from a hat. Data collection from a single participant took approximately 15 min. Once the participant’s loaded data in the experimental group were collected, the participant then crossed over into the unloaded state (control group) and vice versa. There was a 30-min delay between data collection in the loaded and data collection in the unloaded state and vice versa, which allowed the participant to rest. The acute intervention that distinguished experimental and control groups was that the experimental group carried their habitual head-loads. The study measured the participant’s habitual head-load and not their maximal heal-load. The control group, during the unloaded phase, stood without a head-load, while the experimental group carried their habitual head-load.

### Cranial loading musculoskeletal pain-related questionnaire

This study measured the incidence of head-loading-related neuromusculoskeletal pain experienced by the participants using the Ellapen et al. (2009) Cranial Loading Questionnaire, measuring the incidence of head-loading-related musculoskeletal pain among a cohort of female South African youth who carried head-loads on a daily basis. While this questionnaire measured the incidence of musculoskeletal pain incurred from head-loading, the validity and reliability of the questionnaire was not cited in the literature (Ellapen et al. 2009). This study’s proposal, which contained the questionnaire, was presented to a panel of experts in the field of biokinetics and occupational health, who approved the use of the questionnaire (content validity) (reference number REC2020-11-013). The authors furthermore conducted a pilot study (test–retest) to ascertain the trustworthiness of the data collection protocols (intraclass correlation [ICC] *r* = 1.00 reliability). The questionnaire was isiZulu language-edited. Participants were presented with both the English and the isiZulu questionnaire. The participants completed the questionnaire after carrying the cranial load. Furthermore, the participants carried these cranial loads on a daily basis and were able to remember whether their cranial loads caused pain. The mining of data gathered from the cranial or head-loading questionnaire was conducted by an individual proficient in both English and isiZulu. The head-loading questionnaire recorded the mass of the habitual head-load carried, the duration and distance the head-load was transported, as well as the topographical terrain over which the external head-load was transported. The fundamental health-related pain questions were whether the participants experienced pain and discomfort during the transportation of the head-load, as well as the post-health effects of head-loading, the type of pain and the anatomical sites that sustained pain due to head-loading. The questionnaire contained both closed-ended and open-ended questions (Ellapen et al. 2009). A graduate research assistant fluent in isiZulu assisted parents and youth with the questionnaires if queries of uncertainty arose.

### Proprioception measures

Proprioception is the body’s ability to sense movement and its position. Poor proprioception leads to neuromusculoskeletal injury (Prentice 2015). Participants’ proprioception was measured using the portable Biodex stability system (BSS) SD (Biodex System 2) (Biodex Medical Systems, Inc.). All participants started the proprioception test on a stable surface of 10 pins, which was gradually reduced to one pin over a 60-s period, where one pin is the least stable platform. Participants were requested to maintain their optimal balance on the progressively unstable surface for the 60-s duration of the test. The ‘limits of stability’ (LoS) test was employed. The LoS test measures the points at which the person’s centre of gravity approaches the limits of their base of support and then executes a correction strategy in order to return their centre of gravity to within the base of support. The test provides three measures: overall stability index, anterior–posterior (front–back) stability index and medial–lateral (side-to-side) stability index. The overall stability index measures the variance of foot platform displacement in degrees, from level and stable, in all motions during a test as the platform becomes less stable. A high number is indicative of poor overall stability. The anterior–posterior stability index measures the variance of foot platform displacement from a stable level of 10 pins to an unstable level of one pin, where movement occurs in the sagittal plane. A high index reflects poor anterior–posterior stability. The medial–lateral stability index is the measure of the variance of foot platform displacement from a stable level of 10 pins to an unstable level of one pin, where movement occurs in the frontal plane. Participants could hold onto the guard rails if they felt they were going to fall. Furthermore, a graduate research assistant stood behind the participants to catch them if they fell, thereby reducing the risk of injury. No incidents of falls occurred during data collection. The proprioception of participants was measured during the unloaded (control) and head-loaded phases (experimental).

### Statistical analyses

The statistical analyses involved descriptive and inferential analyses. The descriptive analyses included mean, standard deviations and percentage differences. The inferential statistical analyses involved parametric data analyses and nonparametric data analyses. The parametric data analyses involved paired *t*-test and effect size analyses of participants’ head-loaded versus non-head-loading proprioception scores, while the nonparametric data analyses involved chi-square (*X*^2^) analyses of the head-loading neuromusculoskeletal pain questionnaire. The proprioception data fall under the categorisation of continuous data, and scores can range from 0.00 to infinite. The greater the score, the poorer the proprioception of the participant. The *t*-test is commonly used in statistical analysis of continuous data which are normally distributed. The probability level was set at *p* ≤ 0.05.

### Ethical considerations

Ethical approval was obtained from the Tshwane University of Technology (REC2020-11-013).

The following ethical approval procedures were adhered to: ethical approval was obtained: (1) from the Department of Sport, Rehabilitation and Dental Sciences Internal Research Ethics Committee; (2) from the Faculty of Science Committee for Research Ethics; and (3) from the Tshwane University of Technology Research Ethics Committee. The study received the ethical approval number REC2020-11-013. The researchers followed the subsequent steps in order to receive gatekeeper approval: a briefing with the Royal Court of iLembe District was held, explaining the nature of the study and answering all their questions. Upon the approval of the Royal Court of iLembe District (Zondi 2020), the researchers held a briefing session with community leaders (residence chiefs) and the community (parents, guardians and children). Information letters describing the purpose of the study were given to the participants, and the study was furthermore explained to the community (leaders, parents, guardians and children). At the briefing meeting with the community, informed parental consent and child assent documents were handed out to the community. All questions posed by the community were answered. Following this, an intermediator was identified who was present at the time of data gathering so as to ensure that participants were not coerced. The intermediator was a local community leader who was not involved in the study. Prior to data collection, the intermediator collected all parental informed consent and child assent documents. The study followed the South African coronavirus disease 2019 (COVID-19) precautionary guidelines (Greef 2020), as well as the Helsinki Declaration.

## Results

**FIGURE 2:**
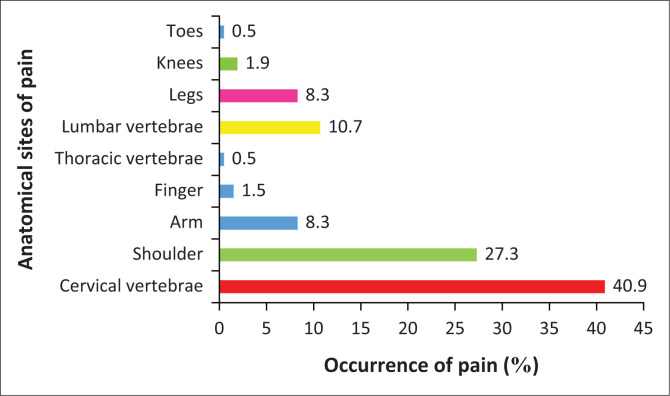
Graphic display of the anatomical sites most vulnerable to cranial loading–induced musculoskeletal pain.

The results of the study will be presented in the following sequence: demographic characteristics (Table 1), musculoskeletal health (Figure 2) and proprioception indices (Table 2) (*n* = 100).

**TABLE 1:** Demographic details of participants (*n* = 100).

Demographic characteristics	Participants
Age (years)	12.3 ± 2.5
Body mass (kg)	44.4 ± 13.7
Stature (cm)	145.0 ± 10
Head-load carried (kg)	8.0 ± 2.5

kg, kilograms; cm, centimetres.

**TABLE 2:** Comparative review of participants’ proprioception when unloaded and head-loaded (*n* = 100).

Proprioception indices	Unloaded	Loaded	*p*-value	Effect size	% diff
Overall proprioception index	3.18 ± 1.87	3.09 ± 1.5	0.728	0.03*	2.83
Anterior–posterior index	2.16 ± 1.78	2.34 ± 1.4	0.226	−0.08	−8.33
Medial–lateral index	1.4 ± 0.81	1.67 ± 0.93	0.031*	−0.21*	−19.28

diff, difference.

* , Indicates significance of the *p*-value and effect size.

The participants’ average body mass index (BMI) was 20.5 kg/m^2^ ± 4.5 kg/m^2^. The ranges of the demographic characteristics were age: 9–17 years, body mass: 23.6 kg – 82.4 kg and stature: 120.0 cm – 168.8 cm. The percentage of external head-load in relation to participants’ body mass was 17.9. This was calculated in equation 1, as follows:

$$\text{External cranial load}=\frac{\text{Mean cranial load }(\text{kg})}{\text{Mean body mas }(\text{kg})}=\frac{8.0\text{ kg}}{44.4\text{ kg}}=17.9\%$$ [Eqn 1]

### Musculoskeletal pain questionnaire analysis

All the participants carried an average head-load of 8.0 kg ± 2.5 kg daily. Participants carried an average external head-load of 2.47 km ± 4.09 km. The path they walked on varied from a flat surface (8.3%) to an uneven surface (31.1%), to hilly terrain (15.4%), to grassy terrain (13.6%), to muddy terrain (3.0%) and to rocky terrain (28.5%) (*p* < 0.0001). The participants began carrying head-loads from the mean age of 8.8 ± 2.01 years. Almost all the participants (96%, *n* = 96) experienced pain when carrying head-loads (*p* < 0.0001). The anatomical sites most susceptible to pain included the cervical vertebrae (40.9%), shoulders (27.3%), lumbar vertebrae (10.7%), legs (8.3%), arms (8.3%), knees (1.9%), fingers (1.5%), thoracic vertebrae (0.5%) and toes (0.5%) (*p* < 0.001) (Figure 1). The reported types of pain experienced when carrying head-loads were dull aching (37.6%), sharp (29.1%), radiating (17.6%), pins and needles (12.1%) and other (3.6%) (*p* < 0.0001). Of the 96 participants who experienced pain when carrying cranial loads, only 66 (68.75%) sought treatment (*p* < 0.0001). Participants used the following three treatments: rest (83.0%), medication (14.2%) and other (2.8%) (*p* < 0.0001).

The overall proprioception index of participants differed negligibly between unloaded and head-loaded states, as indicated by the effect size (*d* = 0.03) (*p* > 0.05). While there was a nonsignificant difference in the anterior–posterior index between the unloaded and head-loaded states (*d* = −0.08) (*p* > 0.05), a significant difference in the medial–lateral index (% difference = −19.28%, *d* = −0.21) (*p* < 0.05) between unloaded as compared to head-loaded states was nevertheless found (Table 2). Skewness of the unloaded versus the loaded proprioception scores in the medial–lateral index is −0.1, suggesting the distribution is approximately symmetric.

## Discussion

The discussion of the results will first consider the impact of head-loading on the participants’ neuromusculoskeletal health and then on their proprioception.

### Musculoskeletal injury profile

Most of the participants who carried head-loads experienced pain, which corroborates previous findings (Echarri & Forriol 2002, 2005; Ellapen et al. 2009; Kurten et al. 2021a, 2021b; Porter et al. 2013). The anatomical sites most vulnerable to pain were the cervical vertebrae, shoulders, lumbar vertebrae and arms, which concurs with the studies of Echarri and Forriol (2002, 2005) as well as with Ellapen et al. (2009) and Geere et al. (2010). Echarri and Forriol (2005), Ellapen et al. (2009) and Geere et al. (2010) reported that most pain was experienced along the porters’ vertebral column, a finding which supports those of this study. The following biomechanical postulations are associated with head-loading vertebral pain: Kurten et al. (2021) reported that head-loading produced a deviated posture manifested in diminished vertex height, which concurred with both the studies by Echarri and Forriol (2002, 2005) and Ellapen et al. (2009). Head-loading compressed intervertebral discs, which subsequently accounted for the diminished vertex height (Echarri & Forriol 2002, 2005; Ellapen et al. 2009; Kurten et al. 2021a), with intervertebral disc compression also being known as intervertebral disc prolapse or herniated nucleus pulpous (Mansfield & Neumann 2014; Prentice 2015). Echarri and Forriol (2005) confirmed that intervertebral disc prolapse caused by head-loading produced severe radiating neuromusculoskeletal pain from the cervical vertebrae to the shoulders and arms. Ellapen et al. (2009) reported that head-loading also produced a change in the craniovertebral angle, which resulted in the anterior translation of the superior cervical vertebrae onto the inferior vertebrae also causing intervertebral disc impingement or compression. This intervertebral disc impingement or compression manifests along the cervical vertebrae, shoulders and arms as paraesthesia (nerve pain) (Mansfield & Neumann 2014). The authors postulated that the aforementioned biomechanical cascade of events does explain the association between intervertebral disc compression precipitated by head-loading and symptoms of paraesthesia from cervical vertebrae to shoulders and arms.

Ellapen et al. (2009) reported that head-loading further produced lumbar intervertebral disc compression, resulting in radiating paraesthesia in the lumbar and sacral vertebrae and legs. Kurten et al. (2021a) reported that head-loading produces anterior pelvic tilt, which hyperextends the lumbar vertebrae producing excessive lumbar lordosis; the head-load causes the lumbar vertebrae to hyperextend, which compresses the lumbar intervertebral discs, producing paraesthesia. Excessive lumbar lordosis is synonymous with paraesthesia of lumbar and sacral vertebrae and of the lower limbs (Mansfield & Neumann 2014; Prentice 2015).

The authors of the present study postulate that head-loading produces a cervico-kypholordotic deviated posture. A cervico-kypholordotic posture is a combination of excessive cervical lordosis, thoracic vertebrae kyphosis and lumbar vertebral lordosis. This postulation is supported by the theory of serial distortions of the human vertebral closed kinetic chain, which suggests that, when one vertebral segment is displaced from its anatomical position, it causes subsequent vertebrae to follow suit, resulting in an amplification of the normal cervical–thoracic–lumbar vertebral anterior posterior anatomical support curving of the human vertebral column (Prentice 2015). Cervico-kypholordotic posture has being recorded among pubescents and adolescents who carried heavy school backpacks (Hundekari et al. 2013), which is also a method of carrying external loads.

The subsequent series of pathomechanical events that participate in a cervico-kypholordotic posture is hypothesised as follows: the external head-load anteriorly rotates the pelvis and hyperextends the lumbar vertebrae, which causes serial distortion up the closed kinetic chain of the vertebral column, resulting in an attenuated posterior thoracic curvature (excessive kyphosis), subsequently leading to an altered craniovertebral angle. The reciprocal relationship between the increased anterior pelvic angle and the altered craniovertebral angle relates to the human body’s natural anterior–posterior weight-bearing engineering curvature (Mansfield & Neumann 2014). When the pelvis is anteriorly tilted, the pubescent could fall forward; thus, in order to prevent a forward fall, the thoracic vertebrae hyper-flex and the cervical vertebrae are drawn either anteriorly or posteriorly, thereby altering the craniovertebral angle. This postulation requires empirical validation by further research. Future research should measure the effect of head-loading on the closed kinetic chain system reviewing cervical, thoracic and lumbar vertebral alignment, standing pelvic rotation, sagittal plane knee alignment (such as genu recurvatum), frontal plane knee alignment (such as genu valgum or varum) and navicular height and drop, in both the sagittal and frontal planes among females who carry head-loads daily in rural regions of Africa.

This study reported that participants simultaneously complained of sharp, radiating pain, pins and needles and dull aching pain, which are suggestive of neural and muscle pathology (Brukner & Khan 2006). The finding of neuromuscular pathology does corroborate the claim that head-loading produces neuromusculoskeletal pain, supporting the previous findings (Echarri & Forriol 2002, 2005; Ellapen et al. 2009). The most widely used treatment method is rest, which corresponds to the findings of Porter et al. (2013). The authors suggest that participants were residents of impoverished rural communities who cannot afford medical treatment and rely on medical staff who operate at rural outreach medical centres. These outreach medical centres do not have sufficient resources to assist patients and are situated a long distance away from residents’ homes. It is postulated that many participants opted to rest their aching bodies given the limited medical assistance they anticipated that they would receive in the rural medical centres. However, it is recommended that deeper investigations be undertaken in order to confirm the aforementioned postulation.

### Comparative proprioception measures between unloaded and head-loaded states

Participants in this study had poor proprioception in the medial–lateral index along the frontal plane, which concurs with the findings of Hoque et al. (1999, 2012), who reported an association between head-loading carriage and poor proprioception, incurring a high incidence of falls and injuries. Proprioception, together with kinaesthetic awareness, helps a person to optimally orientate themselves within their environment, preventing loss of balance (Prentice 2015). The person’s visual, auditory and somatosensation help to maintain optimal positioning within their environment, thereby preventing injury (Prentice 2015). Poor medial–lateral proprioception has been associated with an increased risk of ankle injuries (Han et al. 2015). The most common proprioceptive injury is lateral ankle inversion sprains, where the person’s body mass and/or the external load rapidly moves over the lateral aspect of the weight-bearing ankle in the medial–lateral axis of the frontal plane (Dreyer 2021; Han et al. 2015; Prentice 2015). This pathomechanical event occurs when the ankle rolls laterally (outwards) while simultaneously inverting the foot, tearing the lateral talocrural ligaments (anterior talofibular, posterior talofibular and calcaneofibular) (Gibboney & Dreyer 2021). When one considers the scenario where head-loads are carried while walking through rugged mountain terrain, the possibility of poor medial–lateral proprioception could be responsible for lateral ankle sprains and falls (in the frontal plane), which does adversely affect the quality of life of the rural community dwellers. Krolikowski et al. (2018) and Sanchez-Garcia et al. (2022) reported that mountain hikers sustain a high incidence of ankle and knee sprains (in medial–lateral axis within the frontal plane), which were advocated as being the mechanism of falls. Insofar as the geographic location of iLembe District is in the mountainous region of KwaZulu-Natal, the aforementioned literature does support the premise that poor medial–lateral proprioception stability in the frontal plane could produce ankle pain and falls, causing injury to the lower limbs.

## Conclusion

This study’s findings showed that head-loading adversely affects the neuromusculoskeletal health and proprioception of porters. The anatomical sites most susceptible to neuromusculoskeletal pain were the cervical vertebrae, shoulders and lumbar vertebrae.
